# Elevated Mood Induced by Subthalamic Nucleus Deep Brain Stimulation: A Video-Recorded Case Report

**DOI:** 10.5334/tohm.900

**Published:** 2024-07-12

**Authors:** Patricia B. Coutinho, Kara A. Johnson, Andreea L. Seritan, Nicholas B. Galifianakis, Robert Coleman, Doris Wang, Caroline A. Racine, Jill L. Ostrem, Philip A. Starr, Coralie de Hemptinne

**Affiliations:** 1 Department of Neurology, University of Florida, Gainesville, FL, USA; 2 Norman Fixel Institute for Neurological Diseases, University of Florida, Gainesville, FL, USA; 3 Department of Neurology, University of California, San Francisco, San Francisco, CA, USA; 4 UCSF Weill Institute for Neurosciences, San Francisco, San Francisco, CA, USA; 5 Department of Psychiatry and Behavioral Sciences, University of California, San Francisco, San Francisco, CA, USA; 6 Department of Neurology, Corewell Health, Grand Rapids, MI, USA; 7 Department of Neurosurgery, University of California, San Francisco, San Francisco, CA, USA

**Keywords:** Deep Brain Stimulation, Elevated Mood, Hypomania, Mania, Parkinson’s Disease

## Abstract

**Background:**

Deep brain stimulation (DBS) can be an effective therapy to control motor signs in patients with Parkinson’s disease (PD). However, subthalamic nucleus (STN) DBS can induce undesirable psychiatric adverse effects, including elevated mood.

**Case report:**

We reported a video case of a 73-year-old male implanted with bilateral STN DBS who experienced stimulation-induced elevated mood. A correlation between mood changes and enhanced activation of the ventromedial region in the left STN was observed.

**Discussion:**

This video case report illustrates STN DBS-induced elevated mood and enhances early symptom recognition for patients and diagnostic awareness for professionals.

## Introduction

Parkinson’s disease (PD) is a neurodegenerative disorder characterized by motor symptoms such as tremor, bradykinesia, and rigidity, as well as non-motor symptoms, including depression and anxiety [1,2]. Deep brain stimulation (DBS) is a surgical treatment for medication-resistant PD patients [3] that consists of delivering continuous high-frequency stimulation to the brain through electrodes permanently placed in the basal ganglia nuclei. DBS is an effective therapy for improving motor symptoms and quality of life [4]. However, its impact on non-motor symptoms remains a topic of controversy, with reports indicating both positive effects and potential psychiatric side effects [5]. There are multiple reported cases of mood changes, including (hypo)mania, more recently described as ‘elevated mood’ [6,7,8,9] associated with subthalamic nucleus (STN) stimulation. The term ‘stimulation-induced elevated mood states’ was proposed to describe periods of elevated, expansive, or irritable mood and psychomotor agitation following DBS programming adjustments [7]. These episodes do not meet the Diagnostic and Statistical Manual for Mental Disorders (DSM-5-TR) criteria for mania or hypomania [10]. Increased goal-directed activity, impulsivity, grandiosity, or flight of ideas could be associated with these episodes. This side effect occurs in approximately 4 to 15% of patients following STN DBS for PD [11]. Risk factors include male sex, young onset PD, personal or family history of bipolar disorder, and personal history of dopamine agonist use, impulse control disorders (ICD), dopamine dysregulation syndrome, or substance misuse [7].

Mood changes may depend on the stimulation parameters and fibers activated. Ventromedial stimulation of STN [7,12,13], particularly at amplitudes >3.0 V [14], has been linked to elevated mood states. Although the mechanisms are unclear, positron emission tomography (PET) [15,16,17,18,19] and diffusion tensor imaging (DTI) [20,21] studies suggest that stimulation-induced mood elevation may be associated with modulation of the ventromedial STN or adjacent fiber pathways connecting to limbic circuits, including the dorsolateral prefrontal cortex, the anterior cingulate cortex, and the insula. However, further research is required to investigate the pathophysiology of mood alterations with DBS to better guide contact selection and stimulation parameters and help clinicians prevent this distressing side effect.

Here we report a case of a patient with mood elevation following STN DBS, illustrated with a video recording. Potential anatomical correlates associated with stimulation-induced mood elevation were also studied. Although the clinical aspects of this case were previously reported in a case series ([7], case 1), to our knowledge, this is the first report including a video demonstrating stimulation-induced mood elevation.

## Case Report

The patient was a 73-year-old left-handed man with 7 years of motor signs of PD and no past psychiatric history who underwent staged bilateral STN DBS (Medtronic lead model 3389 attached to two Activa SC pulse generators) for rigidity, tremor, and motor fluctuations (baseline UPDRS-III OFF medication: 43, and ON medication: 30). The patient had no family history of psychiatric or neurodegenerative disease. Preoperative neuropsychological testing revealed mild cognitive impairment secondary to PD, with relative difficulties in executive function, visuospatial skills, attention, and verbal fluency. He endorsed mild-moderate anxiety symptoms, without depression or suicidal ideation. There was mild computer overuse and an episode of levodopa-induced hallucinations and hypersexuality following an increase in his dopaminergic dosage, but otherwise no ICD symptoms. His preoperative medications included: carbidopa-levodopa-entacapone (Stalevo) 37.5-150-200 mg four times daily, benztropine 2 mg nightly, and ropinirole 3 mg twice daily (Levodopa equivalent daily dose (LEDD) 918 mg).

The patient was initially implanted in the right STN. After optimizing the DBS parameters, he experienced significant motor improvement (UPDRS-III ON medication and right STN DBS ON: 15). He was able to discontinue benztropine and reduce the Stalevo dose by half. Five months later, the left STN was implanted, resulting in marked improvement in right-sided motor symptoms. Subsequent programming sessions were challenging due to worsening stimulation-induced dyskinesia at voltages needed to control tremor, despite further weaning of medication by discontinuing ropinirole (LEDD of 100 mg). Over the next year, he experienced three brief episodes of psychiatric symptoms, all associated with activating contact 1 in the left hemisphere (Table 1). The first episode occurred one month after left STN implantation when the patient reported impulsivity at home, feeling ‘hyper’ and ‘dramatic’ after activating a new stimulation group. This episode occurred when parameters were switched to more ventral stimulation on the left STN (from contact C+2– to C+1–) and more dorsal stimulation with interleaving stimulation (ILS) on the right STN (from C+1– to C+1– and C+2–). Symptoms improved by switching back to more dorsal stimulation on the left (C+2–).

**Table 1:** **Deep brain stimulation (DBS) parameters associated with psychiatric symptoms**. STN: Subthalamic nucleus.


TIME AFTER DBS PLACEMENT	SYMPTOMS	SETTINGS ASSOCIATED WITH SYMPTOMS		SETTINGS ASSOCIATED WITH RESOLUTION OF SYMPTOMS	
	
		LEFT STN	RIGHT STN	LEFT STN	RIGHT STN

**6 months**	Impulsivity	C+**1–2.5** V, 60 µs, 140 Hz	STN1: C+1–2.5 V, 60 µs, **125** Hz	C+2–2.6 V, 60 µs, 140 Hz	C+1–2.5 V, 60 µs, 140 Hz

			STN2: **C+2–2.0** V, 60 µs, **125** Hz		

**17 months**	Impulsivity, euphoria and hypersexuality	C+**1–**2–**2.5** V, 60 µs, 185 Hz	STN1: C+1–**3.5** V, 60 µs, 125 Hz	C+2–2.8 V, 60 µs, 180 Hz	STN1: C+1–2.8 V, 60 µs, 125 Hz
	
			STN2: C+3–**2.9** V, 60 µs, 125 Hz		STN2: C+3–2.0 V, 60 µs, 125 Hz

**18 months**	Impulsivity, euphoria and hypersexuality	C+**1–**2–**2.5** V, 60 µs, 180 Hz	STN1: C+1–2.8 V, 60 µs, 125 Hz	C+2–2.8 V, 60 µs, 180 Hz	STN1: C+1–2.8 V, 60 µs, 125 Hz
	
			STN2: C+3–2.0 V, 60 µs, 125 Hz		STN2: C+3–2.0 V, 60 µs, 125 Hz


Ten months later, the patient complained of bilateral worsening of hand tremor (right worse than left), which prompted adjustment of the DBS parameters. The baseline parameters were the following: left STN: C+2–, 2.7 V, 60 µs, 185; right ILS STN1: C+1–, 2.7 V, 60 µs, 125 and STN2: C+3–, 2.9 V, 60 µs, 125. Right ILS was programmed in this patient due to refractory tremor. As part of the programming process, the voltage on contact 1 (C+1–) was then increased to 3.5 V on the right side and changed to double monopolar (C+1–2–, 2.5 V, 60 μs, 185 Hz) in the left STN. The patient had been calm up to this point, with little spontaneous speech. Fifteen minutes after these setting changes, he became agitated and talkative. Encountering the provider, he jumped up and said “I was going to trick you!”. He flailed his arms, clapped loudly, and during gait testing, he spontaneously kicked and walked backward. He could not contain his laughter but denied euphoria and lacked insight into his impulsivity. Reducing stimulation amplitude improved symptoms in the clinic; however, at home, he exhibited impulsive behavior, including an incident at a restaurant where he grabbed a waitress. His behavior was ego-dystonic; he described he was “doing things he wanted to do but also did not want to do.” He consulted a local neurologist who adjusted the DBS settings as follows: left STN: C+2–, 2.8 V, 60 µs, 180 Hz; right ILS STN1: C+1–, 2.8 V, 60 µs, 125 Hz, and STN2: C+3–, 2.0 V, 60 µs, 125 Hz. He reported that the symptoms resolved.

Two weeks later, he returned to the clinic for more detailed testing with video recording to document the patient›s change in mood (Video 1). The Immediate Mood Scaler (IMS), a validated self-report tool for assessing current mood symptoms [22], was used to evaluate acute symptom changes, with higher scores indicating more negative mood states. Additionally, a visual analog scale was employed to measure excitement levels (scored from 0–10). All recordings were done in the afternoon, 6 hours after the patient’s morning medication dose, to limit potential effects of dopaminergic medication. At baseline, he was calm and collected. The baseline IMS was 51, and the excitement scale was 2–3/10. After increasing the amplitude on the right ventral contact (C+1–) to 3.5 V, there were minimal changes in his mood or behavior (IMS 43 and excitement scale 3–4/10). Adding the ventral contact (1–) in the left hemisphere at 2.5 V yielded an immediate behavior change: he felt ecstatic, paced about the room, squatted in a corner, and grabbed a female examiner by the leg. During the neurological evaluation, he stepped backward and swung his arms as though he were conducting an orchestra (IMS –4 and excitement scale 5/10). Reducing the right brain›s ventral contact amplitude to 2.8 V elicited no change (IMS –3 and excitement scale 4/10). After returning to the baseline settings, his behavior changed back to relatively calm, and he reported decreased excitement and impulsivity (IMS 46 and excitement scale 3/10).

**Video 1:** **DBS-induced mood shift**. Activation of contact 1 on the left side elicited immediate euphoric behavior, including pacing, squatting, and grabbing the examiner. Returning to baseline settings resolved the symptoms. DBS: Deep brain stimulation.

**Figure 1:**
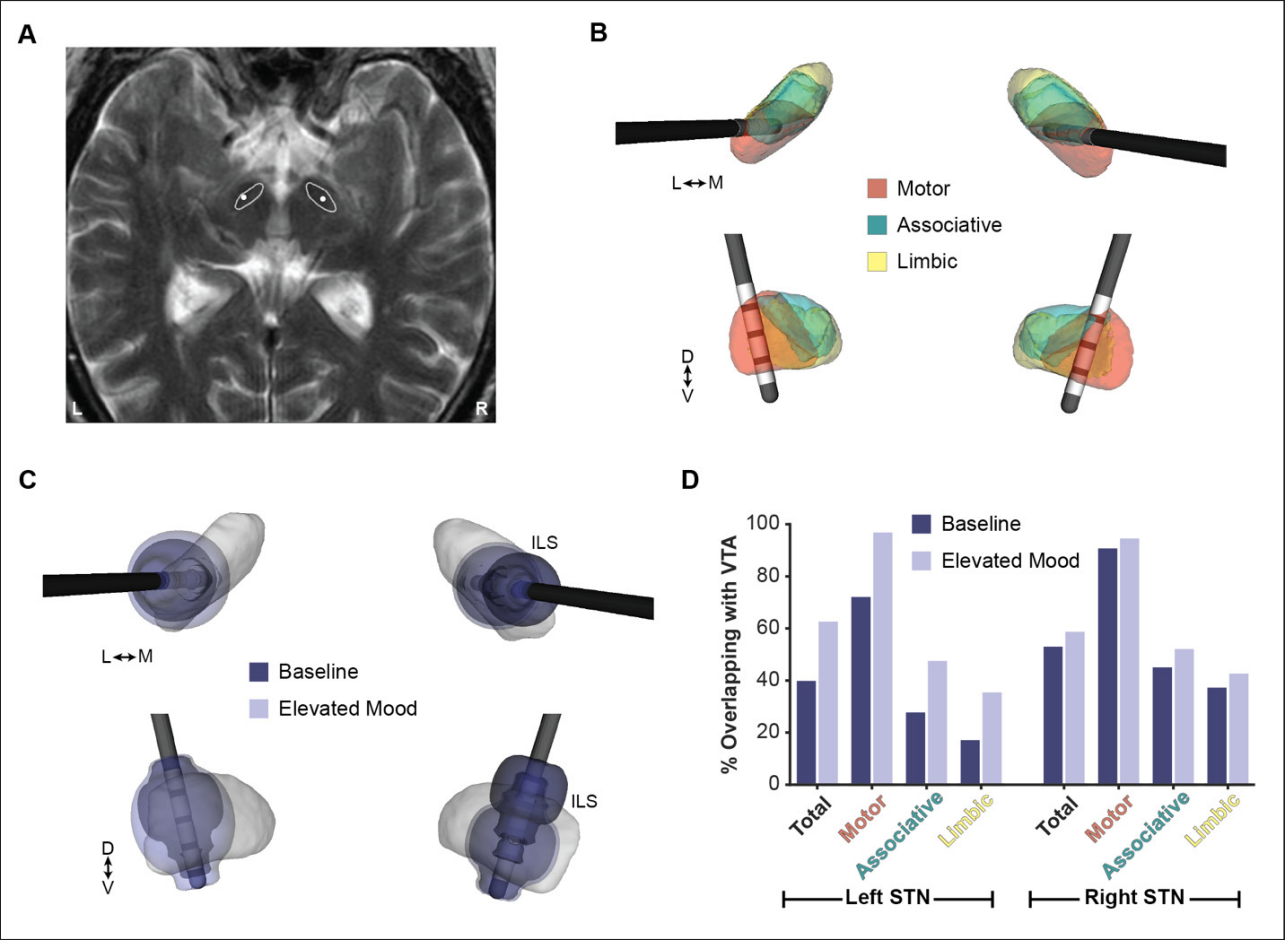
**Imaging analysis of the lead location and stimulation regions that induced elevated mood. (A)** Segmentations of the bilateral STN at the level of contact 1 (marked with white circles) overlaid on the preoperative T2 MRI. **(B)** Bilateral DBS lead locations relative to the limbic, associative, and motor subregions of the STN from the DISTAL atlas (23) nonlinearly registered to the patient’s imaging. **(C)** Models of the VTAs generated by the stimulation parameters that induced elevated mood (light purple) and no mood symptoms at baseline (dark purple). Stimulation of the left ventromedial STN was associated with elevated mood. **(D)** The percentage of the total STN and motor, associative, and limbic subregions overlapping with the VTAs shown in (C). Stimulation parameters that were associated with elevated mood showed higher VTA overlap with the STN and all subregions, with particularly high increases in VTA overlap with the associative/limbic subregions. Right-sided stimulation was less implicated in elevated mood, despite similar levels of VTA overlap with associative/limbic subregions.

Imaging showed that both DBS leads were well placed in the dorsolateral STN (Figure 1A). The DBS leads were localized in the patient’s postoperative T1-weighted MRI. The MNI 2009b nonlinear asymmetric atlas was nonlinearly registered to overlay bilateral segmentations of the STN and respective subregions (motor, associative, and limbic) from the DISTAL atlas [23] (Figure 1B). The volume of tissue activated (VTA) was modeled using established methods [24] to estimate the effects of stimulation on local brain regions. The VTA models indicated that compared to baseline, stimulation that induced elevated mood spread further into the left ventromedial STN (Figure 1C). This spread of stimulation resulted in higher VTA overlap with the motor, associative, and limbic subregions of the left STN. However, the levels of VTA overlap in the left STN were similar to those of the right STN, which minimally impacted mood (Figure 1D). These results suggested that spreading current to limbic/associative region might partly explain the stimulation-induced elevated mood observed in this case.

## Discussion

This study illustrated a case of a 73-year-old male PD patient with STN DBS-induced elevated mood. A video recording depicted the acute shifts in the patient’s mood, complemented by an objective evaluation using a validated scale to assess his emotional state. We concluded that these brief elevated mood episodes were stimulation-induced because they occurred during or shortly after the programming sessions, resolved with setting changes, and were not associated with any other medication changes or medical conditions [7]. ICD is a common comorbidity and risk factor for stimulation-induced elevated mood, and our patient did experience mild ICD symptoms and an episode of levodopa-induced hallucinations and hypersexuality before DBS surgery. However, these symptoms were alleviated by the dramatic postoperative LEDD reduction (by 89%), which was one of the reasons for selecting STN as the DBS target.

STN has distinct anatomical regions related to different functions. The ventromedial area, for example, is linked to the limbic system and emotional regulation [25]. Similar to our case, previous reports have suggested that activation of the ventromedial associative region and medial limbic territory causes STN stimulation-induced elevated mood [7,13,26,27]. The exact pathophysiologic mechanism is still unclear; however, imaging studies have provided insight into the brain networks potentially involved. Studies using PET imaging during STN stimulation have associated stimulation-induced elevated mood with modulation of the limbic circuits, particularly the dorsolateral prefrontal cortex, the anterior cingulate cortex, and the insula [15,16,17,18,19]. Another study found that postoperative impulsivity and disinhibition may be related to greater connectivity between the STN stimulation site and frontostriatal networks, particularly the orbitofrontal cortex [21]. DBS-induced mood changes may also result from stimulation of fiber pathways adjacent to the STN; a previous study utilizing DTI in a patient experiencing stimulation-induced acute mood elevation revealed left coactivation of putative limbic STN tributaries to the medial forebrain bundle (MFB), a pathway linking the medial STN and reward circuitry [20]. Although the majority of studies, including the present case, have implicated the STN, other studies found that stimulation of the substantia nigra might also elicit mood elevation [16,28].

As reported in the previous case series [7], we initially believed that the right STN might have triggered impulsivity in this patient. However, a more detailed analysis of all stimulation settings tested indicated a more significant effect from the left. Imaging revealed a connection between mood change and increased activation, especially of the left STN’s ventromedial area. Right DBS was not associated with significant mood changes, even though it was also stimulating the ventromedial STN with ILS. Our case suggests that mood adverse effects may be lateralized and may depend on the location of stimulation, the specific stimulation parameters used, and personal predisposition.

Similar lateralized results have also been found in other studies [6,20,29]. Imaging analysis of a patient experiencing a stimulation-induced acute hypomanic episode revealed direct contact between one active electrode contact and STN tributaries to the MFB on the left side [20]. In another case report, there was an acute shift in mood from a depressed to a manic state upon turning on the left DBS electrode [6]. However, the laterality of the mood change is not completely established, and other studies found that the right side might also be implicated [16,17].

In our case, the right DBS was programmed with an ILS setting. In ILS, two electrode contacts are rapidly and alternately activated with the same frequency but different amplitudes and pulse widths. ILS enables more targeted stimulation and can minimize side effects and reduce symptoms resistant to traditional stimulation [30]. The use of ILS might account for the lack of observed association between right DBS and elevated mood symptoms. However, further studies are needed to compare the effects of conventional DBS versus ILS on elevated mood symptoms.

Managing DBS-induced mood changes can be challenging. In our case, the episodes of mood elevation were acute, reversible, and associated with changes in stimulation parameters. Clinicians followed the standard of care by adjusting the parameters and reducing dopaminergic medications. If the symptoms had persisted, other strategies might have been considered, including changing stimulation parameters, discontinuing stimulation, pharmacological interventions, and/or psychotherapy, as suggested by Seritan et al. (2021) [7].

This study, however, has limitations. First, to ensure that mood enhancement was solely a left-hemisphere effect, it would have been advantageous to independently test each side while the contralateral DBS was inactivated. However, we modified the two hemispheres’ amplitudes separately, which might have aided in isolating each side. Second, the right side was not tested without ILS. We did not perform these extra tests for ethical reasons because the patient was in significant distress. However, after the first episode of impulsivity, the patient’s DBS was programmed for continuous monopolar stimulation on the right side using the ventral contact (C+1–) at the same amplitude (2.5 V). He remained in this setting for a month with a stable medication dosage and experienced no mood side effects. Future rigorous prospective studies should compare left and right STN stimulation with similar parameters and stimulation regions to adequately determine if this side effect is associated with a specific cerebral hemisphere. Additionally, the VTA is an estimate of the effects of DBS, and current models do not fully account for frequency-specific dynamics such as ILS, which may have played a role in the present case. Atlas-based segmentations were used to identify the STN subregions to illustrate our preliminary hypotheses, but future studies should incorporate connectomic imaging to determine patient-specific structural/functional anatomy. Finally, since this is a single case, our results are preliminary and provide the basis for further research.

In conclusion, our video-illustrated case showed a correlation between mood elevation and increased activation of the ventromedial regions of the left STN. This suggests that reversible mood elevation following STN DBS in PD may be associated with lateralized and stimulation parameter-specific DBS. The medication regimen and stimulation parameters should be individualized to each patient’s motor and non-motor profile, emphasizing the importance of ongoing care with DBS experts. Our findings may help elucidate the neurologic processes underlying hypomania and other mood disorders.
